# Overlapping dose responses of spermatogenic and extragonadal testosterone actions jeopardize the principle of hormonal male contraception

**DOI:** 10.1096/fj.13-249219

**Published:** 2014-06

**Authors:** Olayiwola O. Oduwole, Natalia Vydra, Nicholas E. M. Wood, Luna Samanta, Laura Owen, Brian Keevil, Mandy Donaldson, Kikkeri Naresh, Ilpo T. Huhtaniemi

**Affiliations:** * Institute of Reproductive and Developmental Biology, Department of Surgery and Cancer, and; † Department of Histopathology, Imperial College Healthcare National Health Service Trust, Imperial College London, Hammersmith Campus, London, UK;; ‡ Maria Sklodowska-Curie Memorial Cancer Center and Institute of Oncology, Gliwice, Poland;; § Biochemistry Laboratory, Department of Zoology, School of Life Sciences, Ravenshaw University, Cuttack, India;; ‖ Biochemistry Department, University Hospital of South Manchester, Manchester, UK; and; ¶ Department of Clinical Biochemistry, Imperial College Healthcare National Health Service Trust, Charing Cross Hospital, London, UK

**Keywords:** azoospermia, oligozoospermia, hypothalamic-pituitary-testicular axis

## Abstract

Testosterone (T), alone or in combination with progestin, provides a promising approach to hormonal male contraception. Its principle relies on enhanced negative feedback of exogenous T to suppress gonadotropins, thereby blocking the testicular T production needed for spermatogenesis, while simultaneously maintaining the extragonadal androgen actions, such as potency and libido, to avoid hypogonadism. A serious drawback of the treatment is that a significant proportion of men do not reach azoospermia or severe oligozoospermia, commensurate with contraceptive efficacy. We tested here, using hypogonadal luteinizing hormone/choriongonadotropin receptor (LHCGR) knockout (*LHR*^−/−^) mice, the basic principle of the T-based male contraceptive method, that a specific T dose could maintain extragonadal androgen actions without simultaneously activating spermatogenesis. *LHR*^−/−^ mice were treated with increasing T doses, and the responses of their spermatogenesis and extragonadal androgen actions (including gonadotropin suppression and sexual behavior) were assessed. Conspicuously, all dose responses to T were practically superimposable, and no dose of T could be defined that would maintain sexual function and suppress gonadotropins without simultaneously activating spermatogenesis. This finding, never addressed in clinical contraceptive trials, is not unexpected in light of the same androgen receptor mediating androgen actions in all organs. When extrapolated to humans, our findings may jeopardize the current approach to hormonal male contraception and call for more effective means of inhibiting intratesticular T production or action, to achieve consistent spermatogenic suppression.—Oduwole, O. O., Vydra, N., Wood, N. E. M., Samanta, L., Owen, L., Keevil, B., Donaldson, M., Naresh, K., Huhtaniemi, I. T. Overlapping dose responses of spermatogenic and extragonadal testosterone actions jeopardize the principle of hormonal male contraception.

Testosterone (T) is the crucial hormone for the stimulation and maintenance of spermatogenesis and extragonadal male sexual (*e.g.*, potency and libido) and anabolic (*e.g.*, muscle mass and hemoglobin) functions (1–3). Normally, the intratesticular T (ITT) level is, depending on species, 30- to 100-fold higher than the peripheral T concentration (4–7). The high ITT is considered crucial for the initiation and maintenance of spermatogenesis (8, 9). T is produced in testicular Leydig cells in response to pituitary luteinizing hormone (LH) stimulation, and in the absence of this regulatory link, as found in hypogonadotropic hypogonadism, both testicular T synthesis and spermatogenesis are suppressed.

Suppression of the high ITT by inhibition of LH secretion provides the mechanism of the most widely tested method of hormonal male contraception (8, 10, 11). This is achieved by treatment with exogenous T at doses that slightly increase the normal circulating T concentration, thereby enhancing the negative feedback inhibition of gonadotropin secretion. As a consequence, following LH suppression, testicular T production and ITT concentration decrease below the threshold needed for spermatogenesis. The hypothetical threshold ITT concentration is assumed, but not proven, to be much higher than that needed for the maintenance of peripheral T actions. Since T treatment is used to suppress gonadotropins, peripheral androgen actions persist, and symptoms of hypogonadism can be avoided. This concept has been tested in numerous clinical trials (10, 12, 13), but without uniform suppression of spermatogenesis. A peculiar finding is the wide ethnic variation in contraceptive efficacy (14). While Caucasian men present with 60–70% suppression to azoospermia, the prerequisite for full contraceptive efficacy, reports from China have shown contraceptive efficacy rates of >90% (15, 16). The reasons for the lack of suppression and the ethnic variation therein have been extensively studied, including the obvious lack of complete gonadotropin suppression (17, 18), but so far no mechanism has been found. This outcome has seriously hampered further development of this otherwise promising method of male contraception.

One explanation for the insufficient spermatogenic suppression is that the paradigm of the dependence of spermatogenesis on high ITT, and the maintenance of peripheral androgen actions at much lower concentrations, may not be correct after all. The inhibition of gonadotropin secretion either in contraceptive trials with T (19) or in prostatic cancer patients with gonadotropin-releasing hormone (GnRH; luteinizing hormone-releasing hormone) agonist (5) suppresses ITT by ∼97%, leaving a residual level of ∼50 nM, which is twice the normal peripheral circulating T concentration. As the same androgen receptor mediates androgen actions in the testis and periphery, there is *a priori* no reason why the remaining ITT level could not maintain spermatogenesis.

There is experimental evidence that the T concentration needed to activate spermatogenesis is much lower than normally present in the testis (20), but the doses needed to activate spermatogenesis and extratesticular androgen actions have not been compared. Using the hypogonadal luteinizing hormone/choriongonadotropin receptor (LHCGR)-knockout (*LHR*^−/−^) mouse (21), we earlier showed that qualitatively complete spermatogenesis can be initiated by the basal LH-independent 10 nM ITT level (2% of normal) present in the knockout testes (22). In the present study, we challenged the concept that a dose of T that is able to suppress gonadotropins and to maintain extratesticular androgen effects is insufficient to activate spermatogenesis, which is the prime principle of hormonal male contraception. We treated hypogonadal *LHR*^−/−^ mice with graded doses of T and determined the dose responses of extratesticular and intratesticular T effects. If, indeed, higher T concentration is required for spermatogenesis than for peripheral androgen actions, then the concept of gonadotropin suppression by exogenous T treatment is feasible. If no, or only a slight, difference in dose responses is found, then the concept of hormonal male contraception by T treatment is compromised.

## MATERIALS AND METHODS

### Generation of *LHR*^−/−^ mice

The generation, basic characteristics, and reproductive phenotype of the *LHR*^−/−^ mice were as described previously (21, 22). The mice were maintained under controlled photoperiod (12-h light-dark cycle) at a temperature of 21 ± 1°C, with standard diet and water provided *ad libitum*. All procedures relating to this study were in accordance with the regulations of the UK Home Office Animals (Scientific Procedures) Act and the Imperial College London institutional guidelines for animal care.

### Experimental design of T dose efficacy

To determine the dose responses of peripheral (mating behavior, gonadotropin suppression and anabolic effects) and intratesticular (spermatogenesis) effects of T, 4 groups of *LHR*^−/−^ mice (8–12 mice/group) were implanted between ages 22 and 24 d with 90-d slow-release T pellets of 0.5, 1.5, 2.5, or 5 mg (Innovative Research of America, Sarasota, FL, USA), according to the manufacturer's instructions. An additional higher dose of T was given through subdermal implants of 1-cm Silastic tubing (Helix Medical, Carpinteria, CA, USA), with inner diameter of 1.98 mm and outer diameter of 3.18 mm, filled with T powder (Makaira, London, UK), and sealed at both ends with Loctite Superflex RTV clear silicone adhesive sealant (Henkel, Hatfield, UK). Wild-type (WT) littermates and the *LHR*^−/−^ mice treated with placebo implants were kept as controls for the same period. All surgery was performed under isoflurane (Abbot Laboratories, Maidenhead, UK) anesthesia, with utmost care to minimize suffering. The mice were weighed weekly and their ano-genital distances (AGDs) measured. To avoid dislodging the pellets postsurgery, we assessed the preputial separation, an external sign of male rodent puberty (23), at the end of the treatments (wk 12), shortly before culling.

### Sexual behavior and fertility assessments

The sexual activity of the T-treated mice and controls were assessed as previously described (24, 25). Each male mouse was tested twice; first at wk 6 and later at wk 10 of treatment for sexual behavior during a 30-min test with a WT C57BL/6 female mouse. The females, aged 6–8 wk, were stimulated by the standard superovulation technique with injection of 10 U of pregnant mare's serum gonadotropin (i.p.; Sigma-Aldrich, St. Louis, MO, USA), followed by 5 U of human chorionic gonadotropin (i.p.; Sigma-Aldrich) 46–48 h later. Thereafter, each female was paired with a male. The latency and number of attempted mounts and intromissions over the test period were recorded. Following the test, females were left with the males overnight. Vaginal plugs indicating successful mating were observed early the following morning.

The fertility of the T-treated males and the corresponding controls was assessed after the second sexual activity test at wk 10. This was done by housing them singly with a female of proven fertility until the males were sacrificed at the age of 90 d (3 cycles). Pregnant females were monitored until the birth of litters.

### Evaluation of anabolic effects of T

To assess the effects of T on the body composition, we determined the fat and lean body mass at the end of treatments in nonanesthetized live mice using the rat and mouse quantitative magnetic resonance (QMR) imaging EchoMRI-900 body composition analyzer (Echo Medical Systems, Houston, TX, USA). The measurement allows for serial assessment of fat and lean muscle mass with high precision without any effect on the tested animal. The scans were performed during the light phase of the cycle. Prior to the test, body weight (BW) measurements were taken with an electronic scale. For the procedure, live mice were placed in a thin-walled plastic cylinder (1.5 mm thick, 4.7 cm inner diameter), with a cylindrical plastic insert added to limit movement. The holders were inserted into the QMR machine for a 3-min accumulation scan (26). The data obtained were saved to an integrated computer for subsequent analysis.

### Sample collection and hormone and anabolic parameter measurement

Mice were anesthetized with 2,2,2-tribromoethanol (Avertin; Sigma-Aldrich) by intraperitoneal injection (27) and exsanguinated by cardiac puncture followed by cervical dislocation. Blood samples were allowed to clot overnight at 4°C, after which sera were separated by centrifugation and stored at −20°C until assayed for the peptide and steroid hormones. Testis samples for ITT assay were collected into liquid nitrogen and stored at − 80°C until required.

Serum LH and FSH concentrations were determined by immunofluorometric assays (Delfia; Perkin-Elmer-Wallac, Turku, Finland) as described previously (28, 29). Serum T and ITT were measured with Waters Acquity UPLC and Waters TQS tandem mass spectrometer (Waters, Manchester, UK; ref. 30). For the measurement of ITT, frozen right testes were weighed, decapsulated, and homogenized in PBS. Debris was removed by centrifugation, and the supernatant was used for T measurements (31).

Total hemoglobin concentration was determined from fresh blood using the QuantiChrom Hemoglobin Assay Kit (DIHB-250; BioAssay Systems, Hayward, CA, USA). Serum IGF-1 concentration was determined using Mouse/Rat IGF-1 ELISA kit (R&D Systems Europe, Abingdon, UK) according to the manufacturer's instructions. Total cholesterol, high-density lipoprotein cholesterol, and triglycerides were estimated using the automated Architect ci16200 system (Abbott Diagnostics, Maidenhead, UK). The low-density lipoprotein cholesterol was calculated using the formula of Friedwald *et al.* (32).

### Testicular histology and stereological estimates

After excision, testes were weighed to determine the gonadosomatic index (GSI; *i.e.*, gonad mass as a proportion of total body mass). Thereafter, the left testis of each animal was fixed in Bouin's fluid (Sigma-Aldrich) at 4°C overnight. The fixed tissues were thoroughly washed in water and gradually dehydrated through ascending grades of ethanol to absolute, cleared in histoclear (National Diagnostics, Hessle Hull, UK), and embedded in paraffin wax. Serial sections of 5- to 6-μm thickness were cut, mounted on polylysine microscope slides (VWR, Lutterworth, UK), and dried at 37°C. Sections were thereafter stained with the standard hematoxylin and eosin (H&E) techniques. All photomicrograph assessments were done with the NanoZoomer 2.0-HT digital slide scanner (Hamamatsu Photonics, Welwyn Garden City, UK) and analyzed using the NDPview software (Hamamatsu Photonics).

For stereological estimates, sections from 5–6 animals/group were analyzed. Five slides of sections cut from the middle of the testis were selected, and 3 sections from each slide were analyzed for testicular cell-type composition. Quantitative analyses of spermatogenesis were carried out by counting the number of Sertoli cells, types A and B spermatogonia, and pachytene spermatocytes and round spermatids, steps 1 to 8 (33). The nuclei of different germ cells were counted in 100 round seminiferous tubule cross sections, chosen at random for each mouse. The counts were subjected to Abercrombie correction for section thickness and differences in the nuclear or nucleolar diameter (34), as modified by Amann (35): average number of cells = [number of nuclei counted × thickness (μm) of section]/[average diameter of nuclei (μm) + thickness (μm) of section].

Results of cell numbers per cross section of seminiferous tubule (36) were expressed as the following: coefficient of efficiency of spermatogonial mitosis = (pachytene spermatocytes)/(type A1 spermatogonia); rate of germ cell loss during meiosis (meiotic index) = (round spermatids)/(pachytene spermatocytes); and Sertoli efficiency = (round spermatids)/(Sertoli cell nuclei).

### Quantitation of spermatogenesis

For the average sperm available for release, the density of elongated spermatids (steps 15–19) per testis was counted from photomicrographs of testicular lumens of WT controls and those of T-treated mice showing elongated spermatids. They were counted from the seminiferous tubular sections of 3 randomly selected testicular cross sections of the testis from each animal. The numerical densities of elongated spermatids were averaged across the 3 testicular cross sections assessed to obtain a mean count for each individual animal (37).

### Statistical analysis

One-way analysis of variance (ANOVA) with Newman-Keuls multiple comparison test and Duncan's new multiple range test were used as applicable. The level of significance was *P* < 0.05. Statistical tests were carried out using the Prism 5.0 software (GraphPad, La Jolla, CA, USA).

## RESULTS

### T treatment induces testicular descent and growth of external genitals of *LHR*^−/−^ mice

**Figure 1.**
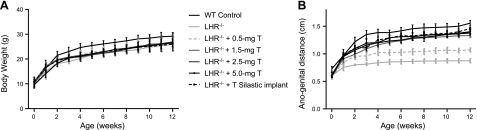
BW (*A*) and AGD (*B*) of *LHR*^−/−^ and T-treated *LHR*^−/−^ mice and corresponding WT controls (8–12 mice/group). BWs of the *LHR*^−/−^ and all T-treated *LHR*^−/−^ mice were significantly lower than those of the WT controls (*P*<0.05), but no differences were found between the T doses. AGD showed significant (*P*<0.05) dose-dependent response to the T treatments.

We observed no significant difference in the mean weights at weaning between *LHR*^−/−^ mice and the corresponding WT littermates. The weekly growth curves for the treated mice over the 90-d treatment period showed no significant difference between untreated *LHR*^−/−^ mice and the different T treatment groups, which were all ∼13% lower than the weights of the WT control mice at the end of treatments (*P*<0.001; **Fig. 1*A***). From the age of weaning (d 20 postpartum), the *LHR*^−/−^ mice exhibited the typical nonmasculinized appearance with feminized external genitals, different from the WT littermates and characterized by shorter AGD, which showed dose-dependent increase in response to T treatments (*P*<0.001), reaching a length similar to those of the WT mice at doses of ≥2.5 mg T (Fig. 1*B* and **Table 1**). Mice treated with doses of 2.5 and 5.0 mg T, as well as Silastic T implants, underwent testicular descent and growth of external genitalia, which were largely indistinguishable from the WT littermate controls.

**Table 1.** Body and organ weights and testicular cell composition of WT control and *LHR*^−/−^ mice and *LHR*^−/−^ mice after treatment with various doses of T

Parameter	WT control	*LHR*^−/−^	*LHR*^−/−^ + 0.5 mg T	*LHR*^−/−^+ 1.5 mg T	*LHR*^−/−^ + 2.5 mg T	*LHR*^−/−^ + 5.0 mg T	*LHR*^−/−^ + T Silastic implant
BW (g)	30.3 ± 0.59^*a*^	27.0 ± 0.23^*b*^	26.9 ± 0.49^*b*^	26.6 ± 0.58^*b*^	25.5 ± 0.68^*b*^	25.0 ± 0.49^*b*^	25.9 ± 0.36^*b*^
Anogenital distance (mm)	15.0 ± 0.25^*a*^	10.8 ± 0.13^*e*^	11.0 ± 0.06^*e*^	12.4 ± 0.15^*d*^	12.9 ± 0.06^*c*^	13.4 ± 0.16^*b*^	13.5 ± 0.16^*b*^
Testis (mg/g BW)	3.4 ± 0.08^*a*^	0.6 ± 0.007^*b*^	0.7 ± 0.02^*b*^	0.7 ± 0.02^*b*^	1.0 ± 0.04^*c*^	2.6 ± 0.07^*d*^	2.8 ± 0.04^*e*^
Epididymis (mg/g BW)	1.19 ± 0.06^*a*^	0^*b*^	0^*b*^	0.38 ± 0.05^*c*^	0.53 ± 0.20^*c*^	1.02 ± 0.15^*a*^	1.25 ± 0.11^*a*^
SV/g BW	0.94 ± 0.06^*b*^	0^*e*^	0^*e*^	0.21 ± 0.05^*d*^	0.41 ± 0.08^*c*^	0.82 ± 0.11^*b*^	1.42 ± 0.09^*a*^
Gonadosomatic index (%)	0.37 ± 0.02^*a*^	0.07 ± 0.003^*c*^	0.07 ± 0.003^*c*^	0.07 ± 0.004^*c*^	0.08 ± 0.005^*c*^	0.26 ± 0.009^*b*^	0.28 ± 0.008^*b*^
Tubule diameter (μm)	197.5 ± 5.2^*a*^	118.5 ± 4.7^*b*^	131.3 ± 9.5^*b*^	137.2 ± 2.6^*b*^	137.5 ± 5.4^*b*^	183.5 ± 8.1^*a*^	188.2 ± 4.4^*a*^
Spermatogonia A (×10^6^/testis)	8.7 ± 3.6	19 ± 7.4	11 ± 4.8	10.6 ± 3.4	6.7 ± 1.2	7.2 ± 1.6	8.7 ± 3.6
Spermatogonia B (×10^6^/testis)	1.99 ± 0.65	2.92 ± 0.76	2.17 ± 0.73	2.25 ± 8.18	2.18 ± 0.64	1.78 ± 0.33	1.72 ± 0.33
Pachytene spermatocytes (×10^6^/testis)	71.5 ± 14.1	58.5 ± 15.9	56.4 ± 9.0	76.1 ± 17.8	54.3 ± 7.2	56.8 ± 13.2	46.7 ± 6.8
Round spermatids (×10^6^/testis)	187.9 ± 45.3^*a*^	7.5 ± 3.8^*c*^	10.8 ± 3.0^*c*^	14.8 ± 6.8^*c*^	29.3 ± 26.3^*c*^	135.4 ± 30.4^*b*^	132.9 ± 39.2^*b*^
Sertoli cells (×10^6^/testis)	14.5 ± 0.2^*a*^	4.1 ± 0.3^*b*^	4.4 ± 0.5^*b*^	4.4 ± 0.2^*b*^	6.7 ± 0.9^*b*^	16.2 ± 0.4^*a*^	14.3 ± 0.3^*a*^
Elongated spermatids (×10^6^/testis)	32.2 ± 4.6^*a*^	0^*c*^	0^*c*^	0^*c*^	6.9 ± 0.8^*b*^	35.0 ± 3.4^*a*^	35.7 ± 5.5^*a*^
Adrenal (mg/g BW)	0.06 ± 0.01^*a*^	0.10 ± 0.01^*b*^	0.10 ± 0.01^*b*^	0.09 ± 0.006^*b*^	0.09 ± 0.006^*c*^	0.09 ± 0.007^*c*^	0.09 ± 0.007^*c*^
Pituitary (mg/g BW)	0.06 ± 0.008	0.05 ± 0.004	0.05 ± 0.004	0.05 ± 0.004	0.05 ± 0.004	0.05 ± 0.004	0.05 ± 0.004
Liver (mg/g BW)	44.9 ± 4.94	37.7 ± 5.85	39.2 ± 4.83	39.1 ± 2.77	41.0 ± 5.71	43.5 ± 5.71	42.8 ± 6.87
Spleen (mg/g BW)	2.45 ± 0.30^*a*^	3.53 ± 0.61^*b*^	3.61 ± 0.37^*b*^	3.38 ± 0.35^*b*^	3.37 ± 0.55^*b*^	2.83 ± 0.48^*a*^	2.91 ± 0.27^*a*^
Kidney (mg/g BW)	5.59 ± 0.37^*a*^	4.64 ± 0.91^*a*^	4.79 ± 0.28^*a*^	4.81 ± 0.20^*a*^	5.14 ± 0.86^*a*^	6.89 ± 1.35^*b*^	7.98 ± 1.03^*b*^

Each group consists of observations on 8–12 mice (mean±sem). Nondetectable results were assigned a value of 0 for statistical analysis. Different superscript letters indicate significant differences between groups (*P*≤0.05).

### T treatments increased testicular and accessory sex gland weights

As described previously (21), adult *LHR*^−/−^ male mice were infertile, with underdevelopment of the testes and hypoplastic accessory sex organs. The testes were cryptorchid and significantly reduced in size, while the prostates and seminal vesicles were undetectable. With T treatments, the testicular weights increased in a dose-dependent manner at doses > 1.5 mg T, attaining the size of the WT mice with 5.0 mg T and Silastic T implants (Table 1). Likewise, the GSI [gonadal wt/BW (%)] increased in a dose-dependent manner in response to T treatment. The accessory reproductive organ weights, including the relative seminal vesicle weights, also increased dose dependently in response to T (Table 1 and Supplemental Fig. S2*A*).

### Restoration of impaired male-type reproductive behavior and fertility

**Figure 2.**
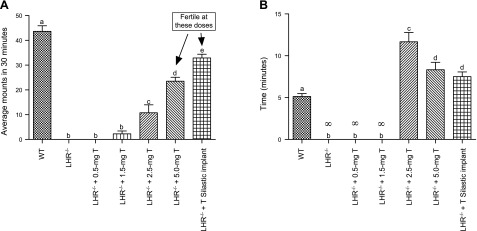
Average + sem number of mounts per male in 30 min (*A*) and mean + sem of mount latency (*B*) in WT control, *LHR*^−/−^, and T-treated *LHR*^−/−^ mice. Different superscript letters indicate significant differences between groups (*P*≤0.05; *n*=8–12 mice/group). ∞, indefinite, no mounts.

The balano-preputial separation, an androgen-dependent marker of puberty, occurred in a T-dose dependent manner and was associated with increased circulating T levels (**Table 2**). The restoration and enhancement of male sexual behavior assessed by the average number of mounts per unit time also occurred in a dose-dependent manner in the T-treated mice (**Fig. 2*A***). The mount latency (Fig. 2*B*) showed that in the untreated *LHR*^−/−^ mice and those treated with 0.5 mg T, mounting behavior and intromissions were completely absent. In mice treated with 1.5 mg T, 1 of 8 mice showed evidence of mounting with a very late latency. Of the 8 mice treated with 2.5 mg T, 4 showed evidence of mounting with intromissions, but none of them sired litters. Further increase in mounting behavior was observed in mice treated with 5 mg T and Silastic T implants, attaining close to 80% of what was obtained in WT mice. These mice also showed evidence of fertility, with 3/8 of mice treated with 5.0 mg T, and 5/8 of those treated with Silastic T implants siring litters. However, the fertility of these T-treated *LHR*^−/−^ mice did not reach the level of the WT males, which all sired litters (Table 2). We found no evidence of embryonic lethality, and the average litter sizes from the fertile 5.0 mg T-treated mice (*n*=5.7) and the T Silastic implant-treated mice (*n*=6.2) were comparable with those of the WT (*n*=7.5).

**Table 2.** Fertility data of *LHR*^−/−^, *LHR*^−/−^ T-treated, and WT control mice

Parameter	WT control	*LHR*^−/−^	*LHR*^−/−^ + 0.5 mg T	*LHR*^−/−^ + 1.5 mg T	*LHR*^−/−^ + 2.5 mg T	*LHR*^−/−^ + 5.0 mg T	*LHR*^−/−^ + T Silastic implant
Males tested	8	8	8	8	8	8	8
Balano-preputial separation	8	0	0	1	6	8	8
Males with evidence of mounting	8	0	0	1	4	6	8
Males ejaculated	8	0	0	0	0	5	6
Fertile males/tested males	8	0	0	0	0	3	5
Females with vaginal plugs/pregnancy	8	0	0	0	0	3	5
Total offspring	57	0	0	0	0	17	31

Fertility of T-treated males was assessed by observing pregnancies in females.

### T treatments increased serum and intratesticular T levels and concomitantly suppressed serum gonadotropins

**Figure 3.**
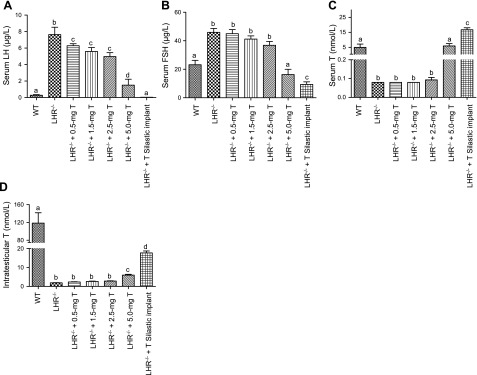
Concentration of serum LH (*A*), FSH (*B*), T (*C*), and intratesticular T (*D*) in WT, *LHR*^−/−^, and and T-treated *LHR*^−/−^ mice. Bars represent averages + se; *n* = 8–12/group. Different superscript letters indicate significant differences between groups (*P*≤0.05).

As expected, serum LH was markedly elevated in *LHR*^−/−^ mice, and it gradually declined with increasing T doses, reaching undetectable levels in mice treated with Silastic T implants (**Fig. 3*A***). The same dose-dependent trend was observed in serum FSH levels, but the suppression was not as profound as with LH (Fig. 3*B*). Serum T concentrations in the nontreated *LHR*^−/−^ mice and in those mice treated with 0.5, 1.5, and 2.5 mg T doses were indistinguishable and near the limit of detection of 0.1 nM (Fig. 3*C*). Dose-dependent increases to the WT range (5.0 mg T) and above (Silastic T implants) were attained with the 2 highest doses. The mean ITT concentrations in control *LHR*^−/−^ mice and those treated with 0.5, 1.5, and 2.5 mg T were similar, and of the order of 1.7–2.7 nM, rising to 5.4 and 17.7 nM in mice treated with the 5.0 mg T and the Silastic T implants, respectively (Fig. 3*D*). These levels however, remained much below the levels of between 120 and 140 nM obtained for the WT littermates.

### Testicular histology, cell composition, and stereological assessments

**Figure 4.**
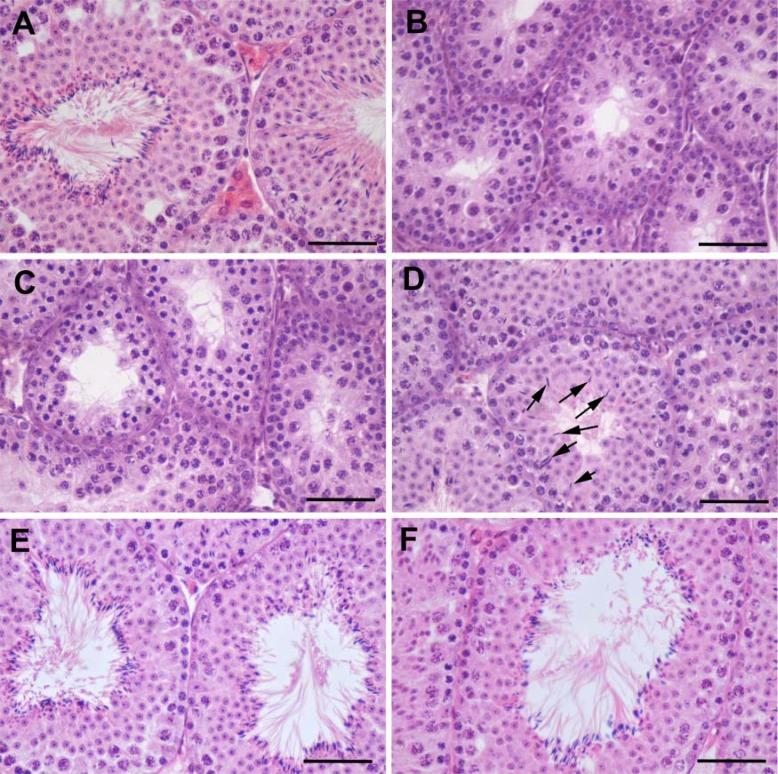
Representative histological images of the testis in WT mice (*A*), *LHR*^−/−^ mice (*B*), and *LHR*^−/−^ mice treated with 1.5 mg T (*C***)**, 2.5 mg T (*D*), 5.0 mg T (*E*), and T Silastic implant (*F*), after 90 d of treatment. Elongated spermatids are clearly visible in the tubules of WT (*A*), 5.0 mg T (*E*), and T Silastic implant-treated (*F*) mice. Arrows (*D*) indicate elongated spermatids in a few seminiferous tubules of the 2.5 mg T-treated mice. Absence of Leydig cells between seminiferous tubules is evident in *LHR*^−/−^ and *LHR*^−/−^ T-treated mice. Scale bars = 50 μm.

The H&E staining of the *LHR*^−/−^ mouse testes (**Fig. 4*B***) and those of the mice treated with 0.5 mg T (data not shown) and 1.5 mg T (Fig. 4*C*) revealed similar cellular architecture. They were marked by narrow seminiferous tubules (Table 1), decreased number and size of Leydig cells, and arrested spermatogenesis at the round spermatid stage, as compared with WT testes (Fig. 4*A*), which showed normal spermatogenesis and formation of seminiferous tubular lumen surrounded by mature elongated spermatids. In mice treated with 2.5 mg T (Fig. 4*D*), progression of spermatogenesis to elongated spermatids was present in a few seminiferous tubules per section of testis (arrowed). In mice treated with 5.0 mg T (Fig. 4*E*) and the Silastic T implants (Fig. 4*F*), numerous elongated spermatids, indistinguishable from the WT testes, were observed in all seminiferous tubule sections.

Stereological analysis of the testes also revealed a significantly lower diameter and proportion of seminiferous tubule lumen in the *LHR*^−/−^ mouse testes. The Sertoli and postmeiotic germ cells demonstrated a marked T dose-dependent rise in absolute numbers (Table 1). At the 2 highest T doses, round spermatid numbers expressed per Sertoli cell were markedly increased and only slightly lower than in WT control mice. The seminiferous tubule diameter, efficiency of Sertoli cells, and calculated meiotic index, all increased in a T-dose dependent manner (Table 1 and Supplemental Fig. S1*A–C*). We observed that mitosis was already restored at low doses of 0.5 mg and 1.5 mg T treatment, with a slight but nonsignificant decrease in trend at 2.5 mg T, and a further increase thereafter at 5.0 mg T and in T Silastic implant animals (Supplemental Fig. S1*D*).

### T treatment increased the density of mature sperm in testicular lumens

To obtain a sensitive measure of the quantity of spermatogenesis, we assessed sperm output by calculating the number of elongated spermatids within testicular seminiferous tubules (Table 1). No elongated spermatids were observed in the lumen of the control *LHR*^−/−^ mice and those treated with 0.5 and 1.5 mg T. Elongated spermatids were observed within the testicular lumen of the 2.5 mg T dose-treated animals, but their density was significantly lower than in the testes following 5 mg T and Silastic T implant treatments, in which the numbers of elongated spermatids per seminiferous tubules were similar to those of the WT controls (Table 1 and Supplemental Fig. S2*B*).

### Anabolic responses to T: hemoglobin, lipid profiles, and lean body and fat mass

There were no significant differences in the hemoglobin levels following treatment with different T doses, and no significant changes were observed in most of the lipid fractions analyzed (**Table 3**). The only lipid parameter with significant response to T treatment was the triglyceride concentration, which decreased to ∼60% of the WT level (*P*<0.05) at the 2 highest doses (Table 3 and Supplemental Fig. S2*C*). Serum IGF-1 levels did not respond to the treatments (Table 3). As expected, fat mass of the treated mice decreased at the 2 highest doses, and lean body mass already increased significantly with the 1.5 mg T-dose implant (Table 3 and Supplemental Fig. S2*D*).

**Table 3.** Anabolic biochemical and body composition parameters

Parameter (*n*=8–12)	WT control	*LHR*^−/−^	*LHR*^−/−^ + 0.5 mg T	*LHR*^−/−^ + 1.5 mg T	*LHR*^−/−^ + 2.5 mg T	*LHR*^−/−^ + 5.0 mg T	*LHR*^−/−^ + Silastic T implant
Hb (g/L)	126.5 ± 5.3	118.8 ± 2.3	115.7 ± 3.2	115.9 ± 1.5	116.2 ± 1.4	116.5 ± 2.4	116.7 ± 3.1
Total cholesterol (mM)	2.1 ± 0.2	1.7 ± 0.2	1.8 ± 0.1	1.7 ± 0.2	1.6 ± 0.2	1.5 ± 0.2	1.6 ± 0.1
Triglyceride (mM)	1.0 ± 0.1^*a*^	0.8 ± 0.1^*a*^	0.7 ± 0.1^*b*^	0.7 ± 0.1^*b*^	0.7 ± 0.1^*b*^	0.5 ± 0.06^*c*^	0.6 ± 0.08^*d*^
HDL-cholesterol (mM)	1.0 ± 0.2	0.8 ± 0.2	0.8 ± 0.1	0.9 ± 0.1	0.8 ± 0.1	0.8 ± 0.1	0.8 ± 0.1
LDL-cholesterol (mM)	0.5 ± 0.1	0.7 ± 0.06	0.6 ± 0.06	0.7 ± 0.1	0.7 ± 0.1	0.6 ± 0.1	0.6 ± 0.1
IGF-1 (nM)	115.3 ± 17.1	93.6 ± 33.5	117.1 ± 24.3	110.2 ± 26.4	106.3 ± 18.4	100.8 ± 26.4	102.9 ± 20.4
Fat mass (g)	5.6 ± 0.6^*a*^	7.3 ± 1.0^*b*^	6.5 ± 1.0^*a*^	6.0 ± 1.0^*a*^	4.8 ± 0.8^*a*^	2.5 ± 0.2^*c*^	3.2 ± 0.4^*d*^
Lean mass (g)	20.4 ± 0.3^*a*^	17.1 ± 0.5^*b*^	17.4 ± 0.4^*b*^	18.6 ± 0.2^*c*^	18.9 ± 0.5^*c*^	19.0 ± 0.5^*a*^	19.3 ± 0.4^*a*^

WT control and T-treated *LHR*^−/−^ mice after 90 d of treatment with T (means+sem; *n*=8–12/group). Different superscript letters indicate significant differences between groups (*P*≤0.05).

## DISCUSSION

We present in this study new findings on the crucial concept of male hormonal contraception: that a single dose of exogenous T is able to maintain the extragonadal androgen actions without simultaneously turning on spermatogenesis. Our observations on the hypogonadal *LHR*^−/−^ mouse model indicated that the doses of exogenous T that effectively suppressed gonadotropins and maintained sexual function also activated spermatogenesis. It thus appears that the therapeutic width of the contraceptive T therapy is very narrow, or even nonexisting, which might explain its insufficient efficacy. While suppressing gonadotropins and ITT production, exogenous T may simultaneously promote spermatogenesis. We also showed that the ITT concentration needed to activate spermatogenesis is considerably lower than the normally occurring high level.

The androgen effects on testicular function have been extensively studied in animal experiments, demonstrating the dependence of immature testis maturation on this hormone, and on subsequent spermatogenesis, T most critically regulates meiosis and spermiogenesis (9). It has become dogma that the high ITT is needed for the activation and maintenance of effective spermatogenesis (8, 9). However, it is not fully understood why the ITT concentration should be so high, because of the facts that the same androgen receptor with similar affinity is functional everywhere in the body and the ITT level is more than one order of magnitude higher than that needed to saturate the androgen receptor (38). High ITT may only reflect the accumulation of T in the testis on its biosynthesis in Leydig cells.

In fact, the need of high ITT for the maintenance of spermatogenesis is largely a conjecture rather than based on solid experimental data. Studies on rodents have shown that reduction of ITT to levels much below the control values can still support spermatogenesis. Cunningham and Huckins (39) first showed that qualitatively complete spermatogenesis can be maintained in rats despite a 30-fold reduction in ITT concentration from control values. In other studies, full spermatogenesis has been maintained at an ITT concentration 30% of normal, while stimulation of spermatogenesis can be observed at ITT concentrations less than 5% of normal (40, 41). Singh *et al.* (6), treating the gonadotropin-deficient *hpg* mice with T, observed that qualitatively complete spermatogenesis was induced without a measurable increase in intratesticular androgen levels but with a dose dependency to blood T levels. In the *LHR*^−/−^ mouse, we have shown previously that qualitatively complete spermatogenesis is achieved in the testes exposed to ∼2% of the normal T level, and this spermatogenesis was T dependent because it could be blocked by antiandrogen treatment (22). It may still be that the high ITT is necessary for qualitatively and quantitatively full spermatogenesis. However, sufficient sperm production to maintain fertility may require much lower exposure, which may be a critical caveat in the current approach to a hormonal male contraceptive. What was lacking until the current study was the simultaneous head-to-head comparison of sensitivities of gonadotropin suppression, maintenance of sexual and anabolic functions and spermatogenesis to treatment with exogenous T.

The extragonadal T effects monitored in this study can be divided into androgenic (sexual behavior, suppression of gonadotropins) and anabolic (body and organ weights and composition, hemoglobin, blood lipids). While the lowest dose significantly stimulating sexual behavior was 2.5 mg T, the steepest increase in this response occurred between 2.5 and 5 mg T. A very similar dose dependence was observed in the feedback suppression of gonadotropins. No effect of the treatments was found on BW, but the distribution of fat *vs.* lean body mass showed clear responses, with the first significant increase in lean mass occurring at 1.5 mg T dose and the reduction of fat mass at 5.0 mg T dose. Anogenital distance responded significantly at 1.5 mg T, and the only lipid parameter responding to T was the suppression of triglycerides at 5.0 mg T. With respect to the spermatogenic parameters including testis weight, sperm density in testis, and tubular diameter, the first significant responses were found at 2.5 mg T dose and the greatest increases occurred between the doses of 2.5 and 5.0 mg T. Hence, we could not detect in the mouse a hiatus between the T doses needed to separate the desired sexual and anabolic effects and the undesired stimulation of spermatogenesis.

One caveat of our study with respect to hormonal male contraception is that we assessed the dose response of T induced stimulation of spermatogenesis in hypogonadism rather than T induced suppression of spermatogenesis in eugonadism. However, critical for both approaches is the concentration of intratesticular T needed for the maintenance of spermatogenesis, whether it is increased to initiate the process or decreased to stop it. Indeed, there is evidence from experimental studies that the initiation of spermatogenesis requires an order of magnitude higher T doses than its maintenance (42), which strengthens our findings and conclusions. Hence, on suppression of existing spermatogenesis a more profound drop of ITT is needed, and the doses of T maintaining extragonadal T actions would undoubtedly exceed those unable to maintain spermatogenesis. The concentration of T in human testis is ∼50 nM following gonadotropin suppression by T or GnRH agonist treatments (5, 19). The residual T concentration in the *LHR*^−/−^ mouse testis was in the nanomolar range. This ITT concentration can stimulate the progression of spermatogenesis from round to elongated spermatids in the *LHR*^−/−^ testis (22), and strong spermatogenesis is evoked when T level exceeds this level in the T-treated mice of this study. It is therefore not unexpected that gonadotropin suppression by T treatment alone in the clinical male contraception studies has not been able to bring about complete suppression of spermatogenesis. In fact, one of the most effective contraceptive regimens has been the combination of T with the antiandrogen cyproterone acetate (43). The men achieving azoospermia with this regimen may in fact have been hypogonadal which explains the efficacy. If eugonadal peripheral androgen levels are maintained, the automatic consequence may be that spermatogenesis is not totally blocked. To achieve more robust suppression of spermatogenesis, the remaining gonadotropin-independent ITT level must be even further suppressed. One possibility is to use inhibitors of the testis specific 17β-hydroxysteroid dehydrogenase type 3 (44, 45), which would block the conversion of androstenedione to T. Another alternative, more difficult to envisage and implement, would be to achieve testis-specific inhibition of androgen receptor function. The present findings provide an explanation why a method solely based on gonadotropin suppression does not achieve uniform spermatogenic suppression.

In summary, we provide in this study experimental evidence that spermatogenesis is not dependent on high ITT levels and that it may not be possible, with a single dose of T, to suppress gonadotropins, testicular T production and spermatogenesis while maintaining extragonadal androgen actions. This observation may explain why the clinical contraceptive trials with T have not been able to achieve uniform suppression of spermatogenesis. Observations on mice are not directly applicable to humans, but the principle tested here is very fundamental in order to ascertain whether the dose response to T is the same or different in the testis and peripheral organs. Our findings are therefore likely to have relevance for further development of hormonal male contraception.

## Supplementary Material

Supplemental Data
