# Supplementary material for: Overlapping dose responses of spermatogenic and extragonadal testosterone actions jeopardize the principle of hormonal male contraception
Source: FASEB J. 2014 Jun;28(6):2566–76. doi: 10.1096/fj.13-249219 (PMC4376501; doi:10.1096/fj.13-249219)
Supplement: Supplemental Data [file supp_28_6_2566__index.html]

Overlapping dose responses of spermatogenic and extragonadal testosterone actions jeopardize the principle of hormonal male contraception — Overlapping dose responses of spermatogenic and extragonadal testosterone actions jeopardize the principle of hormonal male contraception — Supplemental Data 

# Overlapping dose responses of spermatogenic and extragonadal testosterone actions jeopardize the principle of hormonal male contraception

## Supplemental Data

**Files in this Data Supplement:**

- Supplemental Data - (*13-249219SuppData.zip; compressed file 249 KB*)
