# Supplementary material for: Overlapping dose responses of spermatogenic and extragonadal testosterone actions jeopardize the principle of hormonal male contraception
Source: FASEB J. 2014 Jun;28(6):2566–76. doi: 10.1096/fj.13-249219 (PMC4376501; doi:10.1096/fj.13-249219)
Supplement: Supplemental Data [file supp_fj.13-249219_13-249219SuppData.zip › FASEBJ_249219_Oduwole OO et al_Supplemental Fig. 1.pdf]

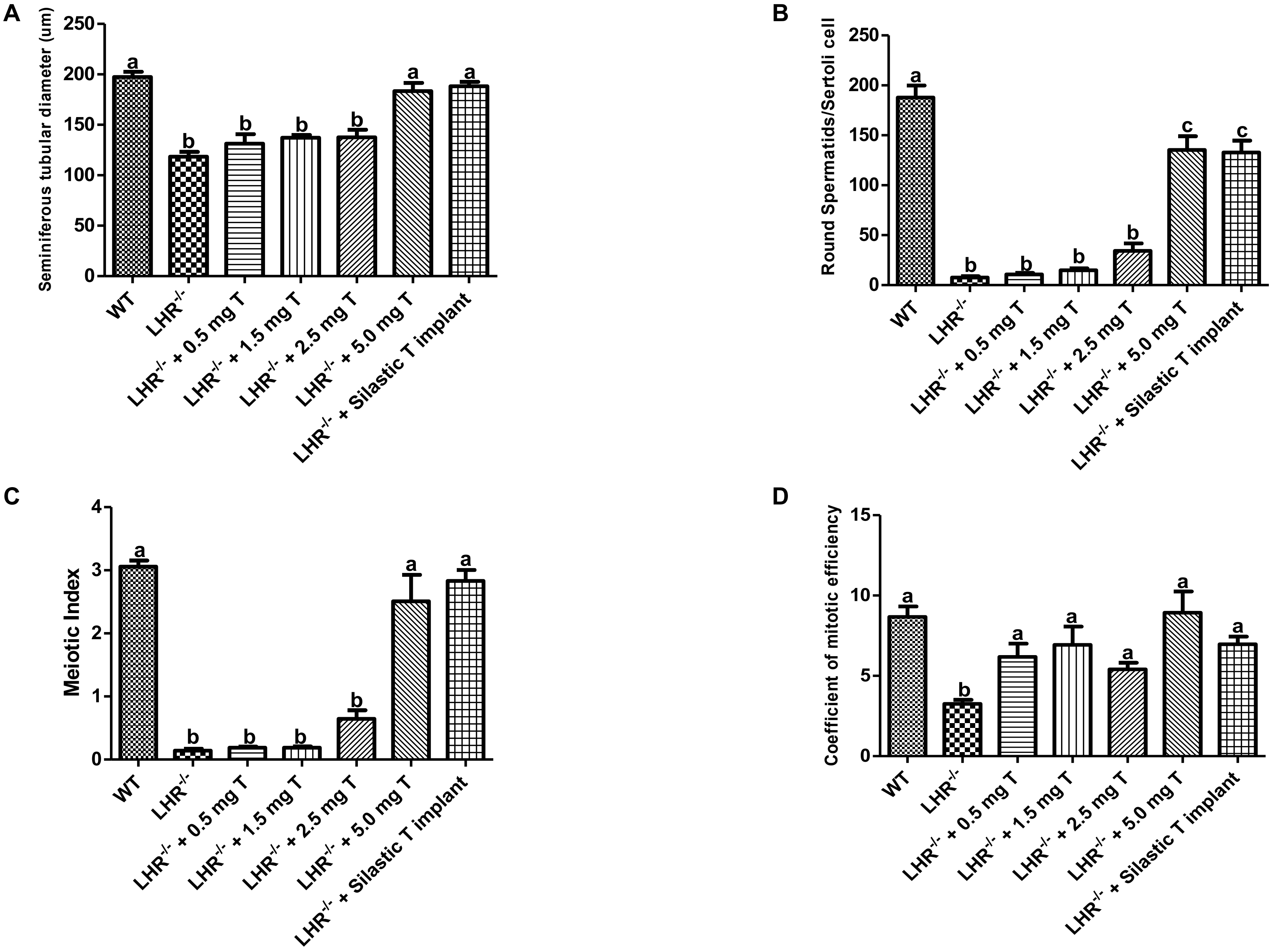

Supplemental Figure 1: Stereological analysis. (A) Semiferous tubule diameter, (B) Round spermatids per Sertoli cells, (C) Meiotic index, and (D) Coefficient of mitotic efficiency of WT control and T-treated LHR$^{-/-}$ mice after 90 days of treatment (mean $\pm$ SEM; n = 8-12 per group). Statistical differences (p at least < 0.05) between the groups are indicated with different superscript letters.
